# Robotic Repair of Acute Traumatic Diaphragmatic Injury From an Abdominal Approach: A Case Report

**DOI:** 10.7759/cureus.68335

**Published:** 2024-08-31

**Authors:** Anna Hargrave, Christian Przeslawski, Carolyn A Solomon Schnurr, Kevin Jamil

**Affiliations:** 1 General Surgery, University of Pikeville, Kentucky College of Osteopathic Medicine, Pikeville, USA; 2 General Surgery, Beaumont Farmington Hills, Farmington Hills, USA; 3 Thoracic Surgery, Corewell Health, Royal Oak, USA

**Keywords:** acute care surgery and trauma, traumatic diaphragm injury repair, minimally invasive repair of diaphragm, robotic repair of diaphragm, traumatic diaphragmatic injuries

## Abstract

Traumatic diaphragmatic injury is a rare condition with a significant mortality risk and may cause a herniation of an intraperitoneal organ into the pleural space. In the acute phase, traumatic diaphragmatic hernia (TDH) may be repaired with laparotomy or thoracotomy and is often associated with multiple concurrent injuries. This case report highlights a rare clinical scenario of blunt traumatic DH in a 62-year-old male with approximately seven centimeters of stomach herniating into the left pleural space, repaired with minimally invasive surgery. This was done via a transabdominal approach with robotic-assisted laparoscopic hernia repair and institution of biologic mesh and represents an important opportunity that potentially reduces the morbidity risk involved with open surgeries.

## Introduction

Traumatic diaphragmatic injuries are a rare occurrence, with an estimated mortality of 18% [1] and an incidence of 0.46% of all trauma cases [2]. This injury may lead to a hernia where an intraperitoneal organ ascends into the thorax after a blunt or penetrating traumatic event [3]. While this is a rare condition, it has been described for centuries, with reports from Sennertus in 1541 [4] and Ambroise Pare in 1579 [5].

Motor vehicle collisions are the most common etiology of blunt traumatic diaphragmatic hernia (TDH) [4]. Ryb et al. found that motor vehicle collisions with lateral crashes and those with changes over 40 km/h are associated with diaphragmatic injuries [6]. The rationale behind why motor vehicle collisions cause blunt traumatic DH is not well understood, but the most accepted hypothesis to explain this injury is that the increased intra-abdominal pressure after impact leads to a substantially higher pressure gradient between the abdomen and the chest. The pressure gradient causes the rupture with intrathoracic herniation and may measure above 100 cm H_2_0, significantly higher than the typical 7-20 cm H_2_O pressure gradient between intraperitoneal and intrapleural pressures [4].

In motor vehicle collisions, the high-energy force that can induce a traumatic DH often causes other life-threatening injuries [4], such as thoraco-abdominal, cerebral, or musculoskeletal injuries [7]. These severe comorbidities are the etiology for the high morbidity and mortality, rather than the traumatic DH injury itself [7].

## Case presentation

**Figure 1:**
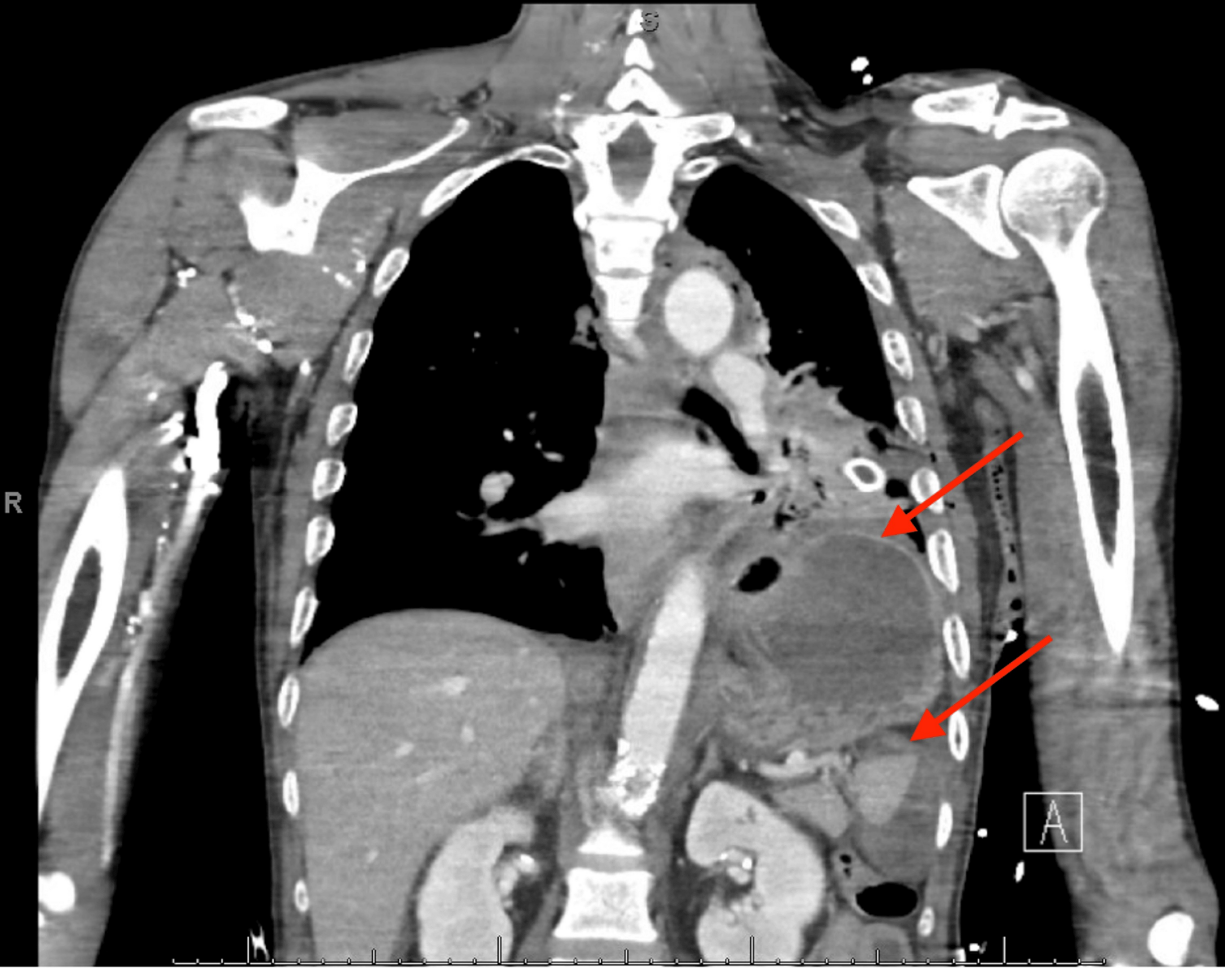
Reconstructed coronal view of chest CT with gastric fundus appreciated within the left pleural space This image shows a traumatic left diaphragmatic hernia measuring 6.9 cm with herniation of the stomach into the thoracic cavity.

A 62-year-old man arrived at a community hospital emergency department unresponsive due to a pedestrian versus motor vehicle collision. Advanced trauma life support (ATLS) protocol was followed, and the patient was intubated for airway protection. He had a left-sided chest tube placed due to decreased breath sounds and became increasingly hypotensive prompting the initiation of a massive transfusion. He had an open-book pelvic fracture confirmed on a secondary survey, which was fixated with an external binder. A chest radiograph was also performed at this time, showing the left chest tube in place without any appreciable herniation of contents into the pleural cavity. He also had bilateral lower extremity deformities that were splinted per the orthopedic surgical team. After stabilization in the trauma bay, the patient underwent a computed tomography (CT) scan with intravenous contrast, revealing a likely diaphragmatic injury with approximately seven centimeters of the stomach, as shown in Figure 1. This injury appeared on the left inferolateral margin of the diaphragm.

Additionally, a small splenic hematoma and a large psoas hematoma secondary to a left pelvic fracture were identified on CT abdominal imaging. Other injuries identified on CT imaging included right frontal subdural hematoma without associated shift, left-sided ribs 7-8 displaced fractures, bilateral rib 4 minimally displaced fractures, suspected left rib 10 minimally displaced fractures, comminuted left clavicular fracture, T4 vertebral body fracture, L1-3 right transverse body fractures, comminuted fracture of left entire pelvis, right superior and inferior pubic rami fractures, and multiple bilateral lower extremity fractures. The injury severity score (ISS) was calculated as 48 out of 75 points, categorizing these traumas as severe chest injury, extremity/pelvis injury, head injury, moderate abdominal injury, and minor face injury.

He was admitted to the surgical intensive care unit for further resuscitation and was later taken to the operating room for bilateral lower extremity washout and placement of external application devices. The patient was hemodynamically stable after this point and was taken by the thoracic surgery team to the operating room on hospital day five for robotic-assisted laparoscopic diaphragmatic hernia repair. This approach was selected due to the associated rib injuries, splenic hematoma, size of the defect, and distorted anatomy.

Past medical history was significant for alcoholism and may have contributed to this trauma, as ethanol level was elevated at 279 on admission. Past surgical history was unremarkable. To surgically correct the TDH, the patient was taken to the operating room, where both his abdomen and chest were prepared using a sterile procedure. Then, five port sites were placed beginning with the camera in a 5-mm port site in the left upper quadrant. Three 8-mm port sites were placed: one at Palmer’s point, one lateral to the left midclavicular line, and one far-right subcostal. An additional port side for a 12-mm assist was also placed to the left of the umbilicus.

On the initial intra-abdominal exam, hepatomegaly and omental adhesions were noted. Once omental adhesions were dissected, identification of left DH was apparent with a herniated stomach protruding through an obvious defect. The stomach was noted to be well vascularized with no apparent perforation. The defect was measured to be 10 x 2 cm. No other obvious intrapleural or intrapleural injury requiring management was observed; however, there were areas of bruising throughout the small bowel and mesentery. To repair the diaphragm, it was approximated with horizontal interrupted mattress stitches using 0 silk sutures with pledgets. A 16 x 20 cm XCM BioLogic® Tissue Matrix biologic mesh (DePuy Synthes, Raynham, MA) was placed over the defect and sutured in place with 0 silk again. The mesh was necessary due to the size of the defect. This mesh was a porcine extracellular biologic matrix, which was chosen over a synthetic mesh due to the patient’s critically ill state and concern for microbial seeding from minute intra-abdominal or intrapleural injuries resulting from the extensive traumatic insult he had sustained.

The patient returned to the surgical intensive care unit intubated and sedated. He remained hemodynamically stable after this point. On hospital day four, the left-sided chest tube was removed. He still required several further orthopedic interventions during this time. Unfortunately, the patient never recovered from a neurological standpoint, and the patient’s family elected to have tracheostomy creation and feeding tube insertion. During the feeding tube insertion, there was no evidence of hernia recurrence. He was eventually transferred to a long-term acute care facility.

## Discussion

This section provides a detailed discussion on the following topics: categorization of the DH, guidelines of the Eastern Association of the Surgery of Trauma, primary repair versus mesh placement, biological mesh versus synthetic mesh, and three similar case reports.

Traumatic diaphragmatic injuries are rare events, but it is important to recognize and intervene in the early stages to reduce morbidity and mortality. To better understand DH, two classification systems exist. The most basic system categorizes DH as acute or chronic. However, there is a discrepancy between the timeline of an acute DH, with some clinicians referring to acute DH as those identified within one week of a traumatic event and others utilizing a one-month cut-off [3,7]. Grimes developed a more precise classification system of three phases (acute, latent, and obstructive). The acute phase refers to the time from the inciting event until the apparent recovery of the injuries that occurred during the inciting event. Once the patient has recovered, the patient enters the latent phase [3]. Patients may be asymptomatic for decades but may present with a severe complication [7], signifying transition to the obstructive phase. This phase occurs when the herniated viscus becomes incarcerated, from which it may become strangulated, leading to necrosis and perforation [3].

Following the classification set forth by Grimes, this case presentation was an acute phase DH. At the time of this robotic intervention, the patient was intubated, with orthopedic interventions still ongoing. By the American Association for the Surgery of Trauma (AAST) criteria, it was grade 3, with the defect measuring 10 x 2 cm.

Additionally, the surgical intervention followed the conditional recommendation from the Eastern Association for the Surgery of Trauma (EAST) published by MacDonald et al. This recommendation was “In stable trauma patients with acute diaphragm injuries, we conditionally recommend the abdominal rather than thoracic approach to repair the diaphragm to decrease mortality, delayed herniation, missed thoracoabdominal organ injury, and surgical approach-associated morbidity (procedural complications, length of stay, surgical site infection, and empyema).” However, the authors acknowledged the limitations of this recommendation because it was based on low-quality evidence. No studies have compared the operative approaches with matched patient populations. This recommendation stems from the historical data on the percentage of repairs performed with either approach and the likelihood of concurrent injuries [2].

An important consideration of DH surgery is a primary repair versus placement of mesh. Based on the evidence from several meta-analyses, reviews, and case series, it is essential to correct the defect with non-absorbable sutures whenever possible. Most surgeons choose to suture two layers with interrupted non-absorbable 2-0 or 1-0 monofilament or braided sutures [8]. This is likely due to the strength and predictability of non-absorbable sutures when compared to absorbable suture types.

With a primary repair correcting a large defect over 3 cm, too much tissue may be lost and may result in excessive tension. A mesh should be placed over the primary repair to decrease the chance of recurrence rate. The recurrence rate with only primary repair has been reported as 42% [8].

Once the decision to use mesh has been made, there are several biological and synthetic mesh types with different utilities. Biological meshes are an attractive option because of the mechanism after implantation. Once this type of mesh is placed, it induces a local inflammatory response [9], gradually breaks down, and promotes host tissue formation with rapid vascularization [10]. The inflammatory response is lesser than that of the reaction of implanted synthetic mesh [10]. Also, biological meshes are associated with lower rates of hernia recurrence and higher resistance to infections [8].

Regarding contaminated fields, there is some controversy on the best option. The contamination can be viewed as a contraindication to synthetic mesh [10]. For example, two cases of a traumatic DH repair were contaminated fields with inflammation of a herniated gallbladder and pleural empyema, and repair was performed with biological implants of human acellular cadaveric dermis or porcine small intestine submucosa. No complications or recurrences were present in the six-month to two-year follow-up period in the two contaminated cases and four other clean cases [9]. The authors noted that this evidence for implanting a biological mesh in a contaminated field was level 5, indicating that further research is needed [9].

However, some surgeons prefer synthetic mesh over biological mesh in clean-contaminated or contaminated DH repairs because of absorbable synthetic mesh’s inability to adhere to the bowel tissue and subsequent reduced risk of bowel fistulation [8]. Currently, there are no studies to assess absorbable synthetic mesh in the emergency setting [8].

Given the mechanism, increased availability, and use of biological mesh in different surgeries, there is a need to directly assess biological mesh versus synthetic mesh. One retrospective study by Lampridis and Billè examined one type of acellular dermal matrix and synthetic meshes in diaphragmatic and chest wall reconstruction. A total of 66 patients reported fewer surgical site complications and readmissions in the biological mesh group than in the synthetic mesh group. However, this study was limited, with a single surgeon, a small sample size, and a short follow-up period [10].

In regards to the specific biological mesh chosen to repair this DH, we elected to use an acellular porcine dermal matrix, the XCM BioLogic® Tissue Matrix (also known as Medeor® Matrix). Based on the results of in vitro studies by Kulig et al., this matrix has a significant impact in terms of cell proliferation and induction of migration, cell attachment, and chemotaxis-driven cell invasion [11]. It was directly compared to three other commercially available extracellular matrices, and there were differences in the type and strength of the immune response from each material. Overall, the XCM BioLogic® Tissue Matrix was the most favorable [11]. Additionally, this extracellular matrix has been studied in chest wall reconstruction in cases with cancer in the chest wall, chest wall deformity or hernia, and chest wall repair following empyema drainage [12].

Reports of robotic repair of DH have been previously published and most often applied to correct chronic or congenital hernias [13]. Additionally, some physicians have utilized this approach in ventricular assist device implantation-associated DH hernias [14]. Three similar case reports have previously been published by Counts et al., Shamim et al., and Holden et al. [15-17]. Each is summarized below.

The case report written by Counts et al. detailed a similar case where a 70-year-old female was presented with blunt traumatic DH due to a motor vehicle collision. However, her rupture and herniation involved the dome of the liver in the right chest. The robotic surgical intervention was performed two months after injury electively because no signs of abdominal injury were present and the patient was clinically stable. These authors utilized an intrathoracic approach [15].

Shamim et al. published a case report describing the laparoscopic repair of left-sided DH from a motor vehicle collision in a 22-year-old patient. A major difference between the cases is that their patient was neurologically intact and hemodynamically stable. Additionally, the colon and stomach had herniated into the left chest. Comparatively, this case report only had stomach herniating into the left chest, but the patient was in a comatose state and had extensive fractures at baseline. The authors chose a laparoscopic transabdominal approach to reduce this patient’s DH [16], differing from this robotic method.

Holden et al.’s publication with an associated video had a unique clinical scenario. The trauma of this case was a prior nephrectomy with complications of inferior vena cava injury. This inferior vena cava injury was repaired through emergent laparotomy and graft reconstruction. The patient presented with upper abdominal pain, shoulder pain, early satiety, nausea, and vomiting. A traumatic DH, measuring 4 x 5 cm, was identified and repaired robotically with an abdominal approach and insertion of a 7 x 11 cm coated polypropylene mesh [17]. Holden’s case with iatrogenic trauma and unique case differs from this presentation, with pedestrian versus motor vehicle accident and unclear deficits due to the patient's comatose state.

This case presentation and publications by Counts et al. [15] and Holden et al. [17] highlight the advantages of the robotic approach. In cases of complex injuries and altered anatomy, the robot offers a superior field of view and ease of maneuverability. Also, it facilitates precise and accurate repairs even in spaces with limited surface area. This minimally invasive aspect poses benefits to both patients and surgeons, with a lower risk of infection when compared to open approaches.

## Conclusions

This case report illustrated a novel application of robotic repair of blunt traumatic DH using an abdominal approach. This patient had a Grade 3 DH according to AAST criteria, with a defect measuring 10 x 2 cm. The defect was successfully repaired with a primary repair followed by biologic mesh, with no complications. Determining the type of mesh was an important consideration, as synthetic and biologic meshes behave differently after implantation. We elected to utilize biologic mesh due to concerns about microseeding. Similar cases were also discussed, demonstrating how rare acute blunt traumatic DH is repaired with minimally invasive methods and how it is a viable option in different scenarios. While this robotic application may not apply to severe acute DH with obstruction, it represents an important modality due to the reduction of complications and anatomic visualization. It should be considered for patients who have acute blunt traumatic DH, with stable hemodynamic and vital signs.
